# Supplementary material for: Genome-Wide ChIPseq Analysis of AhR, COUP-TF, and HNF4 Enrichment in TCDD-Treated Mouse Liver
Source: Int J Mol Sci. 2022 Jan 29;23(3):1558. doi: 10.3390/ijms23031558 (PMC8836158; doi:10.3390/ijms23031558)
Supplement: Supplementary file 1 [file ijms-23-01558-s001.zip › Supplementary_Figure S1.pdf]

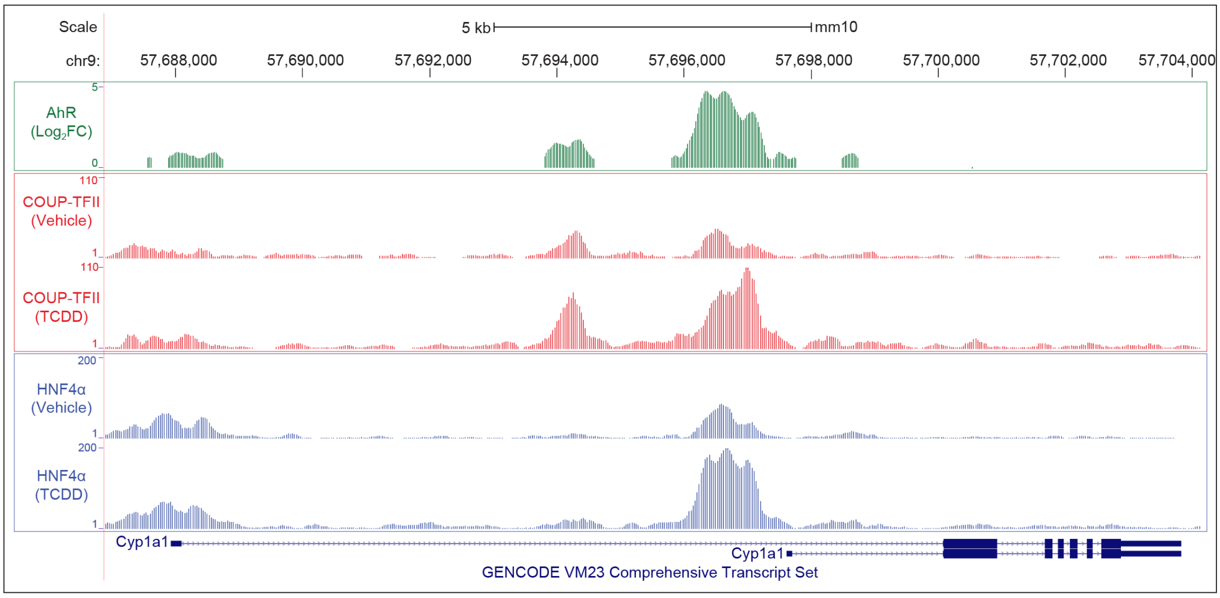

Supplementary Figure S1: TCDD induces differential binding of AhR, COUP-TFII, and HNF4α upstream of the AhR target gene *Cyp1a1*. Differential transcription factor binding was assessed for AhR, COUP-TFII, and HNF4α. Data for AhR shows fold-change of differential binding compared to IgG control following a 2 hr exposure to TCDD in male mouse livers. Genomic binding locations of COUP-TFII and HNF4α are depicted following a 2 hr exposure of either vehicle- or TCDD-treated mice.
